# Influence of Host and Geographic Locale on the Distribution of *Colletotrichum cereale* Lineages

**DOI:** 10.1371/journal.pone.0097706

**Published:** 2014-05-19

**Authors:** Lisa A. Beirn, Bruce B. Clarke, Jo Anne Crouch

**Affiliations:** 1 Department of Plant Biology and Pathology, Rutgers University, New Brunswick, New Jersey, United States of America; 2 Systematic Mycology and Microbiology Laboratory, U.S. Department of Agriculture-Agricultural Research Service, Beltsville, Maryland, United States of America; USDA ARS, United States of America

## Abstract

*Colletotrichum cereale* is an ascomycete inhabitant of cool-season Pooideae grasses. The fungus has increased in frequency over the past decade as a destructive pathogen of *Poa annua* and *Agrostis stolonifera* turfgrass. *Colletotrichum cereale* exists as two lineages, designated clades A and B, but little is known about the distribution of these clades in natural environments, or what role these subdivisions may play in the trajectory of disease outbreaks. In this study, our objective was to determine the frequency of *C. cereale* clades A and B. To rapidly discriminate between the two *C. cereale* clades, a real-time PCR assay was developed based on the *Apn2* gene. A collection of 700 *C. cereale* pathogens and endophytes from twenty Pooideae grass genera were genotyped. 87% of the collection was identifed as part of clade A, 11.7% as part of clade B, and 1.3% was a mixture. *Colletotrichum cereale* from turfgrass hosts in North America were most commonly members of clade A (78%). The overabundance of clade A in turfgrass isolates was directly attributable to the dominance of this lineage from southern sampling sites, irrespective of host. In contrast, 111 *C. cereale* turfgrass isolates collected from northern sampling sites were evenly distributed between clades A and B. Only 28% of *C. cereale* from *A. stolonifera* at northern sampling sites were part of clade A. These data show that environmental factors such as geographic location and host identity likely played a role in the distribution of the major *C. cereale* clades in North American turfgrass.

## Introduction

*Colletotrichum cereale* is a widely distributed fungus that lives in association with monocot grasses of the Poaceae subfamily Pooideae [1], [2]. The fungus inhabits at least twenty cool-season (C3 photosynthesis) Pooideae genera in numerous ecosystems, including cultivated cereal crops, grasses grown for forage, athletic fields and lawns, and natural landscapes such as prairies and grasslands [3],[4]. Although best known as a pathogen of cultivated grasses, *C. cereale* also survives in host tissue without producing any visible signs of disease [2]. *C. cereale* causes anthracnose disease in parasitized grasses, with symptoms varying based on the host and tissue infected [3]. Since the initial description of the fungus in 1908, sporadic but notable disease outbreaks caused by *C. cereale* have been documented [3]. Production of wheat, oats and barley in the United States suffered from severe anthracnose outbreaks during the early part of the 20^th^ century [3]. More recently, grasses cultivated as turfgrass on golf course putting greens have been plagued by destructive anthracnose disease outbreaks, resulting in substantial economic losses and an undesirable but requisite increase in fungicide usage [5]. In turfgrass systems, anthracnose caused by *C. cereale* manifests as either a foliar blight of senescing tissue or a basal stem rot, characterized by blackened, rotted, water-soaked tissue at the base of the plant that eventually leads to host death. Two turfgrass species are primarily affected by anthracnose disease: *Poa annua* and *Agrostis stolonifera* [3].

The emergence of *C. cereale* as one of the primary diseases impacting turfgrass health on golf course putting greens has prompted several investigations in recent years pertaining to the identity of the fungus, the structure of populations, and the management factors that influence the development of disease in turfgrass hosts (*e.g.* [6]–[16]). For most of the 20^th^ century, *C. cereale* was considered conspecific with *C. graminicola*, the fungus responsible for maize anthracnose disease, based on morphological similarities [17]. Multi-locus phylogenetic trees established the uniqueness of *C. cereale* [6], and confirmed the utility of hyphal appressoria as a distinguishing character for *C. graminicola* [18]. Subsequent work showed that *C. cereale* was the basal taxa in a diverse clade of *Colletotrichum* species associated with grasses of the Poaceae family [2], [9], [19]. This assemblage of grass-associated species is collectively referred to as the “graminicola” species aggregate, named after the most prominent member, *C. graminicola* [20]. The graminicola aggregate is populated by at least seventeen species, most of which are limited to just one or a few host species, and infect warm-season (C4 physiology) grasses [2], [21]–[23]. *C. cereale* stands out within the graminicola aggregate for two reasons: (1) this species is the only known member of the group that infects cool-season grasses; and (2) it is plurivorous, with fourteen genera documented as hosts [4].

The wide-host range of *C. cereale* is misleading, as multi-locus sequence analysis shows that the species is subdivided into eleven populations structured according to host/ecosystem origin [2]. The *C. cereale* populations are distributed across two major lineages, designated clade A and clade B [2], [6]–[9]. *C. cereale* clades A and B exhibit an overlapping host range. Both clades are responsible for anthracnose disease in turfgrass, and have also been associated with Pooid grasses as endophytes [2]. Despite substantial evidence for lineage diversification, significant levels of gene flow link clades A and B, indicating that they are of a single species [6]–[8]. Clade A is subdivided into ten subpopulations, each corresponding with a single host (*P. annua* or *A. stolonifera*) or ecosystem (turfgrass, cereal crops, or prairie) [2]. In contrast, clade B is an exceptionally diverse assemblage of isolates from varied environments and hosts, with no subgroups documented [2]. While the subdivision of clade A into subpopulations appears to be driven by host specialization, the factors shaping the earlier diversification of *C. cereale* into clade A and clade B are unknown. Clade A has traditionally been found in higher numbers and encompassing a larger geographic area than clade B isolates [2], [6], [8]. However, this pattern may reflect a bias in sampling rather than structure of natural populations. In this study, our objective was to evaluate a large sample of *C. cereale* isolates to determine the frequency and distribution of clade A and clade B from natural populations, before and after the major turfgrass anthracnose disease outbreaks.

## Materials and Methods

### *Colletotrichum cereale* and other fungal samples

Table S1 summarizes the 700 *Colletotrichum cereale* samples included in the present study. The *C. cereale* samples were derived from four sources: (a) 575 samples were isolates of *C. cereale* established in axenic culture, either new or previously described [2],[6]–[9]; (b) 87 samples were preserved *C. cereale* fungarium specimens consisting of plant tissue colonized by *Colletotrichum* fungi, as diagnosed through the presence of setae; (c) 17 samples were annual bluegrass (*Poa annua*) plants symptomatic for anthracnose disease and showing visible signs of *C. cereale* (setae); and (d) 21 samples were wheat plants (*Triticum aestivum*) asymptomatic for anthracnose disease, but with *C. cereale* setae present on sampled tissue. All wild grown grasses were identified through examination of vegetative features and inflorescences using standard morphological keys for grasses [24]. Species of cultivated cereals and grasses were identified by color, leaf, and visible inflorescence characteristics. Herbarium materials were identified to host following the original collector identifications and confirmed through morphological examinations using the Hitchcock key.

Thirty-four of the cultured isolates were selected to serve as biological replicates. All biological replicates consisted of separate cultures of a given isolate, from which a separate DNA extraction was performed. Ten samples of non-target fungi grown in pure culture were also included as negative controls, along with eight samples of plant tissue (*Viola* sp. and *Poa pratensis*) and two fungarium specimens where *Colletotrichum* was present on dicot hosts (*Cucurma longa*, *Malva rotundifolia*) but for which the species *C. cereale* was not expected.

### DNA extraction and real-time PCR

Genomic DNA from cultured fungal isolates was extracted using a standard phenol:chloroform protocol as previously described [25]. Genomic DNA from fungarium specimens and grass tissue colonized by *C. cereale* was extracted by excising small sections of plant tissue where the fungus was evident (∼5–10 cm^2^), placing the tissue in a 2 ml microcentrifuge tube containing six 2.5 mm glass beads (BioSpec Products, Bartlesville, OK), and shaking in a BioSpec bead-beater (BioSpec Products) on the medium setting for six minutes.

DNA was extracted from the lysed tissue using the Omni Prep DNA Extraction Kit (G-Biosciences, Maryland Heights, MO) according to the manufacturer's protocol; final quantities were assessed using a NanoDrop 1000 spectrophotometer (Thermo Scientific, Wilmington, DE).

**Figure 1:**
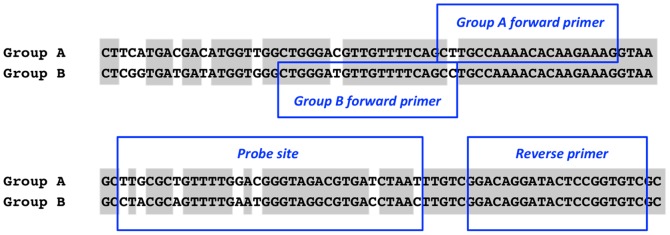
Placement of primers and probes used in real-time PCR. Part of the DNA lyase (*Apn2*) nucleotide sequence used as the template for real-time PCR assay development. Shown is the placement of *Colletotrichum cereale* group-specific forward PCR primers A-Apn2-F, B-Apn2-F with probe sites for *C. cereale* clade A probe A-Apn2 and *C. cereale* clade B probe B-Apn2. Both sets of forward primers and probes utilized the same reverse primer, Apn2-R. Species-specific SNPs that differentiate target lineages are denoted by grey and white shading. Probe binding sites differed between the two clades by seven SNPs.

Target regions for primers and hydrolysis probes for real-time PCR were designed from an alignment of the 750-bp region of the *Apn2* sequencing marker as a template [2]. Primer3 was used to identify optimal sites based on target site DNA properties (http://frodo.wi.mit.edu/primer3/). Two probes, A-Apn2 and B-Apn2, were designed to fall within an 85 or 103 bp PCR amplicon to detect *C. cereale* clade A and clade B isolates, respectively. The two probes differed from one another by 8-bp within the 33-bp probe target site (Fig. 1). The forward primer A-Apn2-F contained one single nucleotide polymorphism (SNP) specific to clade A isolates, and the forward primer B-Apn2-F contained two SNPs specific to clade B isolates. The reverse primer, Apn2-R, was designed for universal use with both forward primers. Primers and probes were synthesized by Integrated DNA Technologies (Coralville, IA); the sequences are summarized in Table 1. Oligonucleotides synthesized for use as probes were modified on the 5′ end with the fluorescent reporter dye 6-carboxy-fluorescein (FAM) and on the 3′ end with the fluorescent quencher dye Iowa Black (IaBk). An additional internal quencher, ZEN, was positioned in the center of both probes to enhance specificity. DNA stocks from cultured *C. cereale* samples were normalized for use in real-time PCR to 15 ng/µl and DNA extracted from leaf tissue colonized by *C. cereale* were diluted 1∶50.

**Table 1:** Real-time polymerase chain reaction primers and dual labeled hydrolysis probes developed in this study for detection of *Colletotrichum cereale* subspecific groups.

Primer/Probe^a^	Description	Sequence (5′ - 3′)
A-Apn2-F	Apn2 forward primer, *C. cereale* group A	CCTGCCAAAACACAAGAAAG
B-Apn2-F	Apn2 forward primer, *C. cereale* group B	CTGGGACGTTGTTTTCAGC
Apn2-R	Apn2 reverse primer, *C. cereale* group A and B	GACACCGGAGTATCCTGTCC
A-Apn2P	Probe, *C. cereale* group A	FAM^b^-TTGCGCTGT-ZEN^c^-TTTGGCGGGTAGACGTGATCTAAT-IaBk^d^
B-Apn2P	Probe, *C. cereale* group B	FAM^b^-CTACGCAGT-ZEN^c^-TTTGATGGGTAGGCGTGACCTAAC-IaBk^d^

a Primer/probe sets reside within *Apn2* locus.

b FAM = 6-carboxy-fluorescein fluorescent reporter dye (IDT, Coralville, IA).

c ZEN =  internal quencher to enhance specificity (IDT, Coralville, IA).

d IBFQ =  Iowa Black Fluorescent Quencher (IDT, Coralville, IA).

The majority of experiments were performed using the Step One Plus system (Applied Biosystems, Foster City, CA); the Roche Light Cycler 480 (Roche Applied Science, Indianapolis, IN) was used for part of the sample. Experiments were performed in 96 well plates: (a) Step One Plus system: MicroAmp Fast Optical 96-Well Reaction Plate (Applied Biosystems); (b) LightCycler 480 White Multiwell Plate 96 (Roche). Reactions consisted of 20-µl volumes containing the following: 2 µl of sample DNA, 1.25 µl of each primer (20 µM stocks), 2.5 µl probe (2 µM stocks), 10 µl Roche Light Cycler 480 Probes Master Mix, and PCR-grade sterile dH_2_O provided with the master mix to volume. The cycling program was as follows: 95°C for 120 s, followed by 45 cycles of 95°C for 5 s, 60°C anneal for 30 s, and 72°C extension for 1 s. All reactions were performed a minimum of three times for all samples.

DNA from *C. cereale* isolates from which the *Apn2* marker was sequenced [2] served as positive controls, and at least one positive control was included with each 96-well plate analyzed. Water blanks were included as negative controls for all plates, and non-target DNA from other fungal species was also tested (Table S2). Positive reactions were scored as those that reached the threshold value prior to cycle 40. Amplification was confirmed for all samples by agarose gel electrophoresis after cycling. Probes were considered specific to *C. cereale* clades if cycle threshold (CT) values were zero for non-target DNA and water controls.

**Figure 2:**
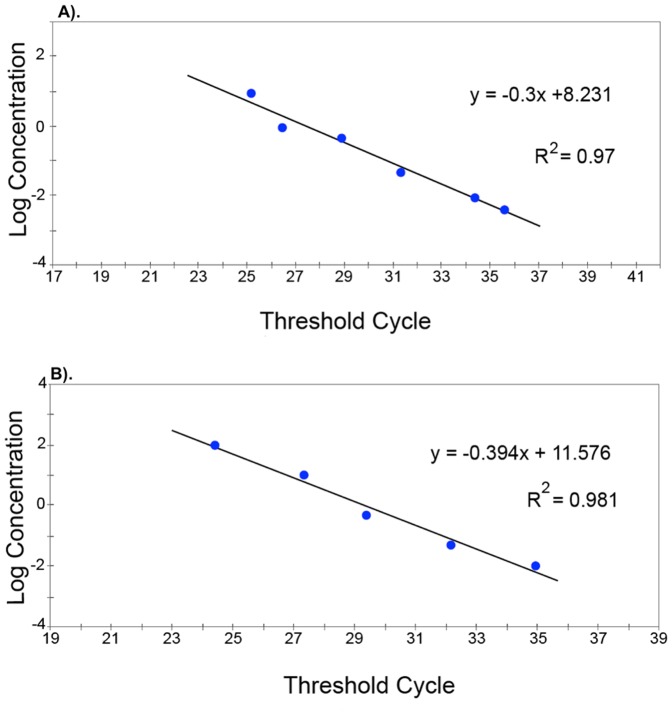
Real-time PCR standard curves. Real-time PCR standard curves showing the linear relationship between the log of known DNA concentrations and the second derivative cycle threshold (C_T_) value. C_T_ values represent positive samples when the fluorescent signal crosses the amplification threshold prior to cycle 40. (a). *C. cereale* clade A probe A-Apn2 with lower detection limit of 4 pg; and (b). *C. cereale* clade B probe B-Apn2, with lower detection limit of 5 pg.

Assay sensitivity was assessed by evaluating two 10-fold dilution curves of genomic DNA extracted from pure cultures of *C. cereale* clade A isolate ANCG 17–15 and *C. cereale* clade B isolate NJ-4990 (Fig 2). Reactions were run on the SmartCycler II (Cepheid, Sunnyvale, CA) using 2.5 µl of each primer (10 µM stocks), 2.5 µl probe (1 µM stock), 5 µl PCR grade water, and Cepheid's Smartmix HM lyophilized PCR master mix. Concentrations ranged from 0.4 pg/µl to 100 ng/µl. Standard curves were generated from the dilutions series data to calculate reaction efficiencies and determine minimum detection levels.

### Statistical Analyses

An exact binomial logistic regression model was fit to the clade distribution data using the logistics procedure in Statistical Analysis System software v. 9.3 (SAS Institute, Cary, NC). The model tested for the effects of region, host species, and the interaction of region and host on clade distribution.

## Results

### Analysis of DNA from cultured *Colletotrichum cereale* isolates

Dual-labeled hydrolysis probes and primer pairs were designed using an *Apn2* nucleotide sequence alignment as a template to identify fixed nucleotide sites that discriminated between the two major *C. cereale* subgroups responsible for anthracnose disease, clades A and B [6]. The lowest level of DNA detection was 4.0×10^−4^ pg and 5.0×10^−3^ pg, for *C. cereale* clade A and clade B, respectively (Fig. 2). Reaction efficencies were 99.53% (amplification factor  = 2) for the clade A assay and 147.74% (amplification factor  = 2.48) for the clade B assay.

**Figure 3:**
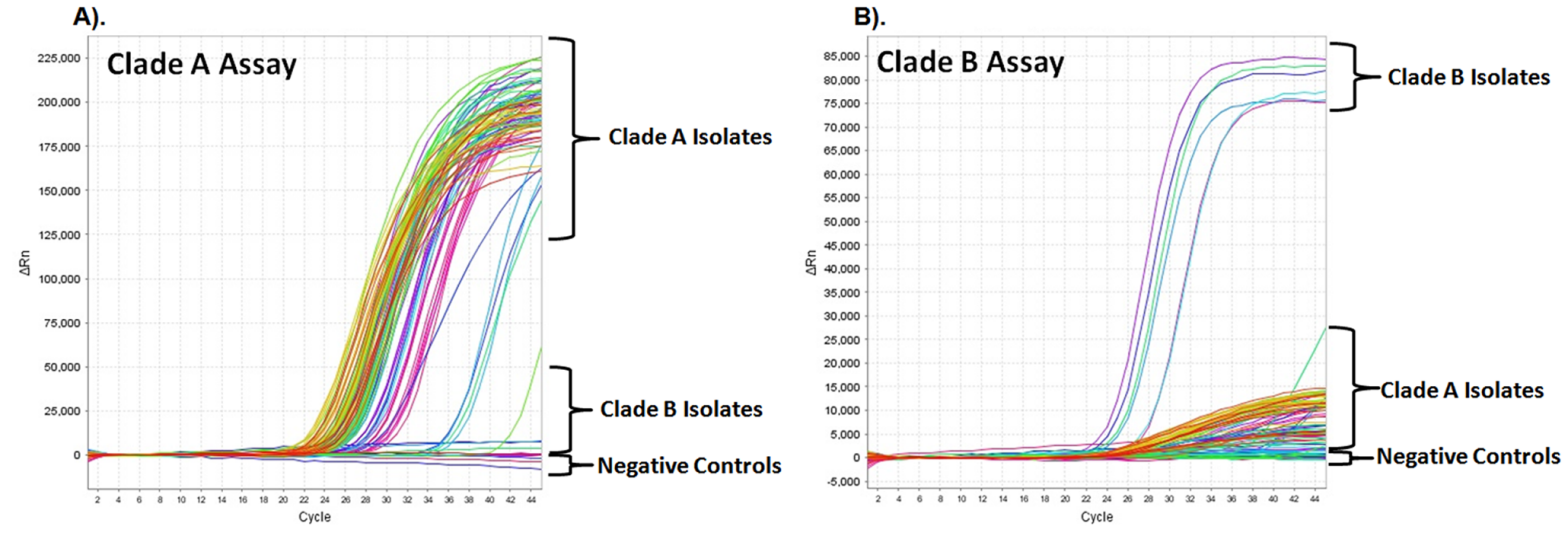
Real-time PCR amplification plots. Real-time PCR amplification plots of *Colletotrichum cereale* probes used to screen a 96-well plate of *C. cereale* infected samples. Negative controls are labeled. Positive controls are included within samples diagnosed as belonging to clade A or B. (a). *C. cereale* clade A probe A-Apn2; (b). *C. cereale* clade B probe B-Apn2.

A sample of 575 cultured isolates of *C. cereale* collected from 20 species of pooid grasses was screened using the *Apn2* real-time PCR assays. 96% of the cultured isolates originated from North America, with 98% of the isolates collected 1998 or later. Data from these reactions are summarized in Table 2 and Table S1. Real-time PCR amplification plots of the *Apn2* probes used to screen a 96-well plate of *C. cereale* infected samples is available in Figure 3. Diagnosis for the 34 samples used as biological replicates yielded the same diagnosis for all replicates (Table S3). Negative controls, water controls and non-target samples produced C_T_ values equal to zero.

**Table 2:** Summary of real-time PCR data generated from *Colletotrichum cereale* samples using *Apn2* detection assay for clade A and clade B.

Clade	Cultured Samples^a^		Fungarium Specimens		*Poa annua* leaf tissue, with visible signs of infection		*Triticum aestivum* leaf sheaths, with visible signs of infection		Total Samples
	Positive Reactions	Avg. C_T_ b	Positive Reactions	Avg. C_T_ b	Positive Reactions	Avg. C_T_ b	Positive Reactions	Avg. C_T_ b	
Clade A	503	27.57	57	35.43	11	27.45	21	26.94	592
Clade A likely^c^	1	38.04	6	37.31	0	−	0	−	7
Clade B	69	27.59	6	35.47	6	28.49	0	−	81
Both Clades	1		8	38.19	0	−	0	−	9
Undiagnosed	1	−	10	−	0	−	0	−	11

700 samples were evaluated, plus ten additional non-target *Colletotrichum* and other species included as negative controls. Second derivative C_T_ (cycle threshold) values represent positive samples when the fluorescent signal crosses the amplification threshold prior to cycle 40. Average C_T_ values are based on a minimum of three technical replicates.

a The 35 biological replicates are not included in the count of positive reactions.

b Average C_T_ values are given as the mean C_T_ generated from all technical and biological replicates.

c Low fluorescence intensity and late C_T_ values (>40.0) were observed for these samples when tested using the B-Apn2 assay.

88% of the cultured *C. cereale* isolates were diagnosed as members of clade A, based on positive calls made using the A-Apn2 assay (avg. C_T_ = 27.57) and negative calls made using the B-Apn2 assay. Twelve percent of the cultured *C. cereale* isolates were diagnosed as members of clade B, based on positive calls made using the B-Apn2 assay (avg. C_T_ = 27.59) and negative calls using the A-Apn2 assay. Analysis of *C. cereale* isolate PA-5018-3, previously shown to possess mixed group A/group B RFLP fingerprints [8], produced a positive diagnosis from both the A-Apn2 and B-Apn2 assays (CT = 25.92 and 24.99, respectively), consistent with the known molecular type for this isolate. From the 609 cultured *C. cereale* isolates, only one isolate (KS-DGI12) could not be diagnosed using either of the two real-time assays. Visualization of the KS-DGI12 reactions using gel electrophoresis showed that no amplicon was produced in any of the reactions (two probes, six replicates). Assessment of KS-DGI12 DNA using the NanoDrop spectrophotometer showed an overabundance of compounds at A230, which may have interfered with the reactions for this sample.

To test the accuracy of clade assignments made using the A-Apn2 and B-Apn2 real-time PCR assays, diagnoses were compared with genotypes of 85 *C. cereale* isolates for which the nucleotide sequence of the *Apn2* locus was already known [2], [6], [8]. 100% of diagnoses made using the A-Apn2 and B-Apn2 assays corresponded correctly with the sequenced genotype of these samples.

### Analysis of DNA extracted from plant tissue

After establishing baseline sensitivity levels and diagnostic accuracy of the clade A and clade B real-time PCR assays using DNA extracted from pure cultures of *C. cereale*, the assays were tested to determine whether *C. cereale* could be detected directly from heterogenous mixtures of fungus and host DNA extracted from *in planta* samples. Data from these reactions are summarized in Table 2 and Table S4.

DNA was extracted from sixteen samples of *Poa annua* maintained in a single 3,344 sq m turfgrass putting green for research purposes [10]. Sample RWCC was obtained from a golf course in northern New Jersey from a *Poa annua* putting green. Plant tissue samples were symptomatic for anthracnose disease; exhibiting overall chlorosis and dark, necrotic stems with visible acervuli present. DNA was extracted from plant tissue where visible signs of the fungus were present within 24 hours of harvest from the field. All seventeen symptomatic plant tissue samples tested positive for the presence of *C. cereale*. Eleven of the samples were identified as members of *C. cereale* clade A (avg. C_T_ = 27.45), and six samples were identified as members of clade B (Avg. C_T_ = 28.49).

The A-Apn2 and B-Apn2 assays were also used to screen for the *in planta* presence of *C. cereale* from 21 wheat samples where *Colletotrichum* acervuli were visible on the leaf sheaths, in the absence of any visible disease symptoms in the colonized host. The wheat samples had been stored at room temperature (∼25°C) for approximately five years before DNA extractions were performed from *Colletotrichum* colonized plant tissue. All 21 wheat tissue samples were positively diagnosed for *C. cereale* clade A (Average C_T_ = 26.94) using the A-Apn2 assay; no clade B diagnoses were made from these samples.

Samples of DNA extracted from plants not known to serve as a host for *C. cereale* (*Viola* sp.) and healthy *Poa pratensis* plants were *C. cereale*-negative when tested with the A-Apn2 and B-Apn2 assays.

### Analysis of DNA from fungarium specimens

After establishing that the A-Apn2 and B-Apn2 real-time PCR assays could be used to detect *C. cereale* groups from heterogeneous mixtures of DNA derived from host tissue colonized with *Colletotrichum*, DNA was extracted from 87 *C. cereale* fungarium specimens and tested using the assays. Data from these reactions are summarized in Table 2 and Table S5. Fungarium specimens consisted of leaf tissue samples from eighteen Pooid grass species ranging from 70–120 years old. Visible morphological signs of *Colletotrichum* – melanized setae – were observed on all specimens through examination using a stereomicroscope. After DNA extraction from the host/fungal matrix, gel electrophoresis showed DNA was fragmented, 200-bp or less, consistent with standard degradation profiles described for ancient DNA samples (data not shown).

*C. cereale* was detected from 88.5% of the 87 fungarium specimens. Average C_T_ values for fungarium samples were 35.43 for clade A (n = 57), and 35.47 for clade B (n = 6), averaging 7–8 cycles later than from DNA extracts from cultured *C. cereale* isolates. Six fungarium specimens were diagnosed as likely belonging to clade A after visual inspection of the amplification curves, as low fluorescence intensity and late C_T_ values were observed for these samples when tested against the B-Apn2 assay. Eight fungarium samples produced positive C_T_ values from both the clade A and clade B assays. Sequence analysis of the resultant amplicon revealed the presence of both the clade A and clade B genotype in these samples (data not shown). The remaining ten fungarium samples identified as *C. cereale* produced no C_T_ values for either clade A or B, as did the non-target species *Colletotrichum capsici* (BPI 397265) and *Colletotrichum sp.* (BPI 397277). Visualization of the real-time PCR product through agarose gel electrophoresis and the amplification product of PCR reactions performed using primers alone on the negative fungarium samples yielded no detectable amplicons.

### Sample-wide frequency of *C. cereale* subspecific groups

Of the 700 *C. cereale* samples, 98.4% were diagnosed using the A-Apn2 and B-Apn2 real-time PCR assays. All but one of the eleven undiagnosed samples were fungarium specimens. Of the 689 *C. cereale* with a group diagnosis, 87% of the sample was part of clade A, 11.7% was part of clade B, and 1.3% of the sample produced mixed A/B diagnoses (Table 2).

Table 3 summarizes *C. cereale* membership relative to host origin as diagnosed using the *Apn2* real-time assays. Overall, *C. cereale* clade A samples were observed from all 32 Pooid host species (Table 3). In contrast, *C. cereale* clade B samples were only observed from eight of the Pooid genera.

**Table 3:** Summary of the diagnosis of *Colletotrichum cereale* subgroups by host species using the *Apn2* real-time detection assays, A-Apn2 and B-Apn2.

	Cultured Isolates		Fungarium Specimens		*In planta* Samples	
Host Species	Clade A	Clade B	Clade A	Clade B	Clade A	Clade B
*Aegilops cylindrica*	1	0	−	−	−	−
*Agropyron repens*	−	−	1	0	−	−
*Agrostis* spp.^a^	49	34	4	−	−	−
*Ammophila arenaria*	1	0	−	−	−	−
*Anthoxanthum odoratum*	−	−	1	0	−	−
*Arrhenatherum elatius*	−	−	2	1	−	−
*Avena sativa*	6	0	9	0	−	−
*Axoponus affinis*	1	0	−	−	−	−
*Bromus* spp.^b^	54	0	7	1	−	−
*Calamagrostis* spp.^c^	29	0	1	−	−	−
*Dactylis glomerata*	72	8	0	2	−	−
*Elymus* spp.^d^	86	0	−	−	−	−
*Festuca* spp.^e^	2	0	−	−	−	−
*Holcus lanatus*	1	0	1	0	−	−
*Hordeum* species^f^	−	−	2	0	−	−
*Phleum pretense*	−	−	2	2	−	−
*Poa* spp.^g^	165	28	1	1	11	6
*Polypogon fugax*	1	0	−	−	−	−
*Secale cereale*	−	−	22	5	−	−
*Triticum* spp.^h^	39	0	18	2	21	0

Biological replicates and negative controls are not included. Dashes indicate that no samples originating from a given host plant were evaluated.

a *Agrostis* species  = *A. alba*, *A. canina*, *A. stolonifera* and *A. tenuis*.

b *Bromus* species  = *B. inermis*, *B. rigidus* and *B. secalinus*.

c *Calamagrostis* species  = *C. acutifolia*, *C. epideios* and *C. inexpansa*.

d *Elymus* species  = *E. canadensis* and *E. virginicus*.

e *Festuca* species  = *F. elatior* and *F. rubra*.

f *Hordeum* species  = *H. jubatum* and *H. vulgare*.

g *Poa* species  = *P. annua* and *P. pratensis*.

h *Triticum* species  = *T. aestivum* and *T. vulgare*.

98% of the *C. cereale* samples in this study originated from within North America. Of the sixteen samples from other geographic locales (Japan, Germany, Australia), all were cultured isolates. Only two *C. cereale* samples from outside North America, CBS 303.69 and CBS 304.69 collected from *Agrostis tenuis* and *Ammophila arenaria*, respectively, in Germany during 1967, were diagnosed by the *Apn2* real-time assay as members of clade B. This diagnosis was consistent with previous nucleotide sequence data for these isolates [2].

The largest component of the *C. cereale* sample was drawn from cultured isolates of the fungus from the two primary economic hosts in North America – the turfgrasses *Agrostis stolonifera* and *Poa annua* (n = 78 and n = 191, respectively). Table 4 summarizes the distribution of *C. cereale* from these two hosts in North American samples. The 269 *C. cereale* samples from turfgrass hosts were plant pathogenic isolates, collected from diseased putting greens between 1998 and 2006, after the emergence of the destructive anthracnose disease outbreaks that took place beginning in the mid 1990s [5]. When this collection of *C. cereale* turfgrass isolates was typed using the *Apn2* assays, the overall ratio of clade A to clade B isolates was 3.6 to 1 (A = 210; B = 59). 87% of all *P. annua* isolates were typed as members of clade A. In contrast, only 56% of the *C. cereale* isolates from *A. stolonifera* hosts were typed as members of clade A.

**Table 4:** Summary of the diagnosis of *Colletotrichum cereale* subgroups by geographic locale using the *Apn2* real-time detection assays, A-Apn2 and B-Apn2.

	*Agrostis stolonifera*		*Poa annua*		Total	
State/Province	Clade A	Clade B	Clade A	Clade B	Clade A	Clade B
**Southern region**						
Alabama, USA	4	0	−	−	4	0
California, USA	−	−	96	1	96	1
Mississippi, USA	6	0	−	−	6	0
North Carolina, USA	12	1	27	2	39	3
Tennessee, USA	4	0	−	−	4	0
Texas, USA	1	0	−	−	1	0
Virginia, USA	4	0	1	−	5	0
**Total: Southern region**	31	1	123	3	154	4
**Northern region**						
British Columbia, CA	−	−	1	0	1	0
Connecticut, USA	2	4	5	1	7	5
Massachusetts, USA	2	3	1	4	3	7
New Brunswick, CA	−	−	1	0	1	0
New Hampshire, USA	−	−	1	0	1	0
New Jersey, USA	−	−	15	3	15	3
New York, USA	1	1	4	1	5	2
Ontario, CA	7	24	2	2	9	26
Pennsylvania, USA	0	1	11	9	11	10
Rhode Island, USA	1	0	2	2	3	2
**Total: Northern region**	13	33	43	22	56	55
**Total, all North America**	44	34	166	25	210	59

Diagnoses are given for isolates of *C. cereale* made from diseased turfgrass hosts *Agrostis stolonifera* and *Poa annua*. Diagnoses are separated according to the geographic locale where the sample originated, broken down by states in the United States of America and provinces in Canada. States/provinces are subdivided into regions according to minimum extreme temperature of location following the USDA-ARS Plant Hardiness Zones. Southern region sampling sites were defined as those sites that fell within USDA-ARS Plant Hardiness Zones 7a to 9a (minimum extreme temperature range −17.8 to −15°C and −6.7 to −3.9°C, respectively). Northern region sample sites were defined as those sites that fell within USDA-ARS Plant Hardiness Zones 6a or colder (minimum extreme temperature range −23.3 to −20.5°C or less). Biological replicates and negative controls are not included. Dashes indicate that no samples originating from a given host plant were evaluated.

The dataset of North American turfgrass isolates of *C. cereale* was evaluated according to two broad geographic subdivisions – designated north and south, according to average annual minimum temperature extremes (Table 4). Analysis of clade A and clade B frequencies in these two broadly defined geographic regions showed that the overabundance of clade A isolates across the entire sample from turfgrass hosts was attributable to the *C. cereale* subsample from the southern region. The 158 *C. cereale* isolates collected from southern sites were predominantly members of clade A; only 2.5% of southern isolates were members of *C. cereale* clade B. In contrast, the 111 *C. cereale* isolates sampled from northern sites were almost evenly divided between clade A and group clade B. However, the frequency of the two *C. cereale* subgroups differed between isolates made from *A. stolonifera* and *P. annua*. Northern region isolates of *C. cereale* made from *A. stolonifera* (n = 46) were most commonly members of clade B by a factor of 2.4 to 1, whereas isolates made from *P. annua* (n = 65) were most commonly members of clade A by a factor of 1.6 to 1.

A clade × region interaction was detected using logistic regression analysis (Table 5). In the southern region of North America, no differences in sampling response were detected among hosts (p = 0.79); there was a 96.9% and 97.6% probability of obtaining a clade A isolate when sampling from *A. stolonifera* or *P. annua*, respectively. However, when sampling in the northern region, there was a 28.3% probability of obtaining a clade A isolate from *A. stolonifera*, whereas there was a 66.1% chance of obtaining a clade A isolate when sampling from *P. annua* turf.

**Table 5:** Exact logistic regression results on the probability of samples diagnosed as *Colletotrichum cereale* clade A across regions and turfgrass hosts.

	Logit	Odds	Probability	*P* Value
**Southern region**				
*Agrostis stolonifera*	3.4337	30.9911	0.9687	0.7916
*Poa annua*	3.7136	41.0012	0.9762	0.7916
**Northern region**				
*Agrostis stolonifera*	−0.9316	0.3939	0.2826	<0.0001
*Poa annua*	0.6702	1.9546	0.6615	<0.0001

## Discussion

The primary objective of this study was to examine the frequency of *C. cereale* clades A and B in the natural environment from a large sample of modern and historical specimens from North America. Our results show that clade A is the predominant group in natural populations of *C. cereale*. Furthermore, the frequency with which we observed clade A from historical specimens indicates that clade A has been the dominant *C. cereale* group in North America for at least a century. Despite the abundance of *C. cereale* clade A in the environment, clade B isolates were also identified throughout the entire sample, as part of both modern collections and from cereal crops and grasses dating back to the original 1908 *C. cereale* fungarium specimens. Thus, on the recent time scale, both lineages are endemic to North America, with direct evidence from fungarium specimens documenting their presence in the United States for over a century. This finding is consistent with the high levels of diversity observed for both *C. cereale* clades from previous multi-locus haplotype analysis [2].

*Colletotrichum cereale* isolates diagnosed as clade A dominated the overall collection screened in this study, comprising 87% of our samples. When the distribution of clades was evaluated based on host origin, we observed a similar trend for all non-turfgrass *C. cereale* isolates, with clade A outnumbering clade B on all non-turfgrass hosts. Of particular note was the broad host range of *C. cereale* clade A, with isolates from this lineage identified from 32 different Pooid grass species. In contrast, *C. cereale* clade B was only identified from eight Pooid species. On *Aegilops, Agropyron, Ammophila, Anthoxanthum, Axoponus, Festuca, Holcus, Hordeum, Phleum*, and *Polypogon* hosts, the dominance of clade A could be a result of limited sampling on these hosts, as each host was only represented by one to four samples. Still, the number of clade A isolates on the remaining hosts greatly outnumbers clade B and should not be discounted. Previous haplotype analysis suggests clade A may be the ancestral group for *C. cereale* [6], and the low frequencies and reduced host range from which we observed clade B isolates supports this theory. While additional data is need to confirm the haplotypes present in our sample collection, it is possible that clade B is transitioning to a broader host range.

The overabundance of clade A observed from the overall *C. cereale* collection did not hold true for isolates obtained from turfgrass hosts (*A. stolonifera, P. annua*) when the samples were subdivided based on host origin and broad geographic range. In southern regions of North America, clade A turfgrass isolates accounted for 97.5% of the sample, regardless of the host. In contrast, in northern regions, *C. cereale* clade A and clade B isolates from turfgrass were found in equal numbers. However, closer examination of the northern *C. cereale* isolates showed that the frequency of clades A and B in turfgrass populations may be host dependent. 77% of *C. cereale* isolates from *P. annua* in the northern region were clade A, whereas 60% of the isolates obtained from *A. stolonifera* were clade B. This data, combined with the results from logistic regression analysis, suggests that there is likely a host preference among turfgrass pathogenic isolates of *C. cereale* in northern regions of North America, and warrants future investigation. Several other graminicolous *Colletotrichum* species are known to exhibit host specificity (*e.g. C. graminicola*, *C. sublineola*, *C. navitas*). It is possible that we may be observing the transition to host specificity among lineages of *C. cereale* as previously hypothesized [2].

The distinct frequencies observed from *C. cereale* clade A and clade B turfgrass isolates based on geographic region is an interesting finding, and rigorous fine-scale sampling should be conducted to confirm this theory. Regardless, we cannot ignore that this apparent geographic distribution may also be influenced by temperature and attributed at least in part to the environmental adaptations of the host. *P. annua* does not tolerate heat stress well [26], therefore it may serve as an opportunistic host for *C. cereale* clade A isolates in southern regions where the host is exposed to significant environmental stresses and weakened prior to infection. The optimum temperature for growth of *C. cereale* in culture and on detached leaf assays has been reported to be anywhere between 22°C to 28°C [27]–[31], whereas optimal infection in the greenhouse has been reported between 15°C–30°C [27], 27°C–33°C [32], and 30°C–33°C [33]. To date, a consensus temperature optima for *C. cereale* has not been determined, likely due to difficulties in establishing a repeatable, greenhouse-based inoculation protocol [5] but also possibly because differences in temperature optima for the two clades were not assessed in previous studies. Varying temperature preferences between *C. cereale* lineages could explain the difficulties surrounding the development of an inoculation protocol, and the range of temperatures reported from early studies, and should be investigated. While temperature extremes seem to play an important role in the *C. cereale* pathosystem, we cannot rule out that other factors in the regions, such as soil type and weather events, may be impacting pathogen distribution. Fine-scale, rigorous sampling is needed to further examine these factors.

Our data shows that clade A and B can co-exist together, as mixed infections on individual plants, or on closely situated plants comprising a single stand of grass. Nine samples derived from a single lesion possessed mixed A/B genotypes. Likewise, of the sixteen samples taken from a single *P. annua* research putting green, ten samples were diagnosed as members of clade A and six samples as clade B. On golf course putting greens, *P. annua* is best known for its ability to colonize established *A. stolonifera* stands, resulting in putting greens of mixed host composition. Given the ability of both clades to exist together in a single putting green, combined with our finding that clades A and B seem to exhibit a host preference in northern regions, golf course putting greens comprised of both *A. stolonifera* and *P. annua* may provide a unique host environment for *C. cereale*. This habitat may allow both clades of the fungus to come in frequent, close contact with one another, potentially resulting in new, aggressive strains of the fungus.

The presence of both major *C. cereale* clades in close proximity to one another – either on the same plant, or within a single stand of grass – raises interesting questions about the interactions between these two lineages, the mechanisms involved in sustaining gene flow while at the same time maintaining the distinction between lineages [2]. The sexual cycle for *C. cereale* has never been documented [3], yet gene flow is known to have occurred between clades A and B, and between several populations of this species [2], [6]–[8]. Several other *Colletotrichum* species (*e.g. C. acutatum, C. lindemuthianum*) are known to complete a parasexual cycle that creates diversity and yields new, pathogenic fungal races [34], [35], thus it is possible that *C. cereale* clade A and B isolates may be undergoing a similar process. This would provide a unique evolutionary advantage to pathogenic *C. cereale* isolates, particularly in heavily managed golf course putting greens, where fungicide resistant strains of *C. cereale* are emerging [36]–[38].

In the current study, we have developed an important assay for the rapid and accurate genotyping of *C. cereale* clades A and B, without the need for time consuming and labor intensive culturing. The biallelic fixation of the targeted A-Apn2 and B-Apn2 probe site for over 100 years demonstrates that utility of *Apn2* as a diagnostic marker for *C. cereale* lineages. This assay provides an accurate, lineage specific identification of *C. cereale* in as little as 45 minutes from DNA extracted directly from infected host tissue. The assay is quantitative, and sensitive enough to detect as little as 4 pg of *C. cereale* DNA from heterogeneous mixtures of host and environmental DNA. This sensitivity and rapid diagnosis is in stark contrast to traditional culturing methods that require surface sterilizations and multiple sub-culturing steps on antibiotic media to eliminate contaminating organisms before yielding a pure culture. From an applied standpoint, this assay could be utilized for clinical diagnoses, to assess pathogen levels from golf course greens, to ensure that seed is pathogen-free, or as an experimental tool to confirm the identity of *C. cereale* clades. For *C. cereale*, we can now use this assay to look at the distribution of clades A and B within a single putting green and to monitor this distribution over time to gain insight about the trajectory of recent anthracnose disease outbreaks in turfgrass.

To our knowledge, this study also marks the first application of real-time PCR experiments for the detection of *Colletotrichum* species from preserved fungarium specimens. The *Apn2* real-time PCR assays were able to reproducibly detect *C. cereale* from small portions of fungarium materials ranging in age from 70 to 120 years old with a high level of success. Previous work with *Colletotrichum* pathogens of warm season grasses – *C. sublineola*, *C. echinochloae*, and *C. caudatum* – has demonstrated the power of DNA-sequence based approaches to accurately genotype type specimens from fungarium materials >100 years old [21], [23]. Our experiments demonstrate the utility and sensitivity of real-time PCR as a tool to conduct molecular examinations of historical fungal collections for other *Colletotrichum* species. Real-time PCR assays are particularly well suited for fungarium specimens, as they rely on amplification of very short regions of DNA. Since post-mortem degradation and shearing of DNA into fragments <500-bp is ubiquitous in historic specimens [39], real-time PCR is well suited to the requirements of working with fungarium materials. The development of similar assays for other *Colletotrichum* species where species concepts are currently in a state of flux [20], may prove useful for typification, species delineation, and the examination of temporal changes in these organisms.

## Supporting Information

Table S1 Cultured Samples of *Colletotrichum cereale* tested to determine clade membership (A or B) using real-time PCR assays. CT = cycle threshold. ^a^Isolate was diagnosed as belonging to clade A following visual inspection of amplification curves. Low fluorescence intensity and late CT values (>40.0) was observed for this sample when tested using the B-Apn2 assay. ^b^Isolate PA-50183 produced a positive diagnosis from both A-Apn2 and B-Apn2 assays, consistent with RFLP fingerprint data [8] previously generated for this isolate. ^c^No diagnosis could be made using either assay. Analysis with the NanoDrop spectrophotometer showed an overabundance of compounds at A230.
(XLS)

Table S2 Samples of non-target fungal species used as negative controls for *Colletotrichum cereale* real-time PCR assays. CT = cycle threshold.
(XLS)

Table S3 Summary of biological replicates of cultured samples of *Colletotrichum cereale* tested to determine clade membership (A or B) using real-time PCR assays. CT = cycle threshold.
(XLS)

Table S4 Fresh samples of plant tissue tested to determine presence or absence of *Colletotrichum cereale* and clade membership (A or B) using real-time PCR assays. CT = cycle threshold.
(XLS)

Table S5 Herbarium specimens of *Colletotrichum cereale* tested to determine clade membership (A or B) using real-time PCR assays. CT = cycle threshold. ^a^Isolate was diagnosed as belonging to clade A following visual inspection of amplification curves. Low fluorescence intensity and late CT values (>40.0) was observed for this sample when tested using the B-Apn2 assay. ^b^Sequence anaylsis of the amplicon generated confirmed the presence of both genotypes in these samples (data not shown). ^c^Visualization of the real-time PCR product through gel electrophoresis and the amplification product of conventional PCR reactions performed using the primers alone on the fungarium specimens yielded no detectable amplicons.
(XLS)
